# A Hypothesis Testing Based Method for Normalization and Differential Expression Analysis of RNA-Seq Data

**DOI:** 10.1371/journal.pone.0169594

**Published:** 2017-01-10

**Authors:** Yan Zhou, Guochang Wang, Jun Zhang, Han Li

**Affiliations:** 1 College of Mathematics and Statistics, Institute of Statistical Sciences, Shenzhen University, Shenzhen, China; 2 College of Economics, Jinan University, Guangzhou, China; 3 College of Economics, Shenzhen University, Shenzhen, China; Universidade Federal do Rio Grande do Sul, BRAZIL

## Abstract

Next-generation sequencing technologies have made RNA sequencing (RNA-seq) a popular choice for measuring gene expression level. To reduce the noise of gene expression measures and compare them between several conditions or samples, normalization is an essential step to adjust for varying sample sequencing depths and other unwanted technical effects. In this paper, we develop a novel global scaling normalization method by employing the available knowledge of housekeeping genes. We formulate the problem from the hypothesis testing perspective and find an optimal scaling factor that minimizes the deviation between the empirical and the nominal type I error. Applying our approach to various simulation studies and real examples, we demonstrate that it is more accurate and robust than the state-of-the-art alternatives in detecting differentially expression genes.

## Introduction

In recent years, next-generation sequencing methods, for instance, ChIP-seq and RNA-seq, due to their distinct advantages in increasing specificity and sensitivity of gene expression, they have become a poular choice in biological studies. Such sequence-based methods have evoked a wide range of novel applications, for instance, splicing variants [1, 2] and single nucleotide polymorphisms [3]. Specifically, RNA-seq has become an attractive alternative to microarrays in the inference of differential expression (DE) between several conditions or tissues, for it gives more accurate detection and measure of gene expression.

We first map the RNA-seq reads to the reference genome and then summarize them as “counts”. That is, we use a count number to measure the expression level of each gene. Under different conditions/tissues, the experiments will result in different total read counts, that is, sequencing depths. In order to make the expression levels of genes comparable and further conduct differential expression analysis, normalization is a crucial step in data processing. The normalization step aims to adjust the systematic technical effects and reduce the noise on the data as well.

Considering the essential difference in technology between microarray and RNA-seq, we can not normalize RNA-seq data with the normalization methods of microarray data directly. A conventional way of RNA-seq analysis is to standardize the data between samples by scaling their total number of reads to a common value. More sophisticated normalizaiton methods could be divided into two groups, which are referred to as the library size concept adjustment and distribution of read counts adjustment. In the first group, several researchers have developed some normalization methods such as modifying the mean expression of a gene with a global factor [4–7]. For instance, Hoen et al. [8] used the square root of scaled counts to analysis LONGSAGE-seq data, and Mortazavi et al. [9] modified sequencing data to reads per kilobase per million mapped (RPKM). Robinson et al. [10] proposed a scale normalization method (TMM), which is a weighted trimmed mean of log-ratios between the test sample and the reference sample. However, TMM method could not normalize the data satisfactorily well for asymmetric data, especially, for large proportion of DE genes. Zhou et al. [11] used an iterating median fold changes to estimate the scale factor and showed that it is more rubust than the TMM method for asymmetric data. In the second group, the standard procedure is to first compute the proportion of each gene’s reads relative to the total number of reads in each library. Assuming there are similarities between the distributions of read counts, the methods [12] [13] match the distributions of all genes across all libraries, either on a single quantile or on all the quantiles. Nontheless, the RNA repertoires may change diversely under different experimental conditions, thus the proportions of gene expressions are not comparable in such case. Some authors proposed to use the housekeeping genes as pivot points and match the distributions of those housekeeping genes, instead of all of genes, for inter-sample normalization. Bullard et al. [13] used a single housekeeping gene in normalization. Chen et al. [14] proposed a method to select a subset of housekeeping genes by analyzing experimentally related GO terms and the stability of gene expression.

The key of normalization problem is to choose an appropriate metric of expression to compare across samples. We propose a novel normalization method by exploiting the knowledge of housekeeping genes. We address the problem from the hypothesis testing perspective, by matching the observed and the nominal false discovery rate. The estimated normalization scaling factor is expected to be stable for different confidence level in the hypothesis testing. Thus it can normalize the samples without trimming the data and avoids the problem of the TMM like methods.

The remainder of the paper is organized as follows. In Section 2, we propose a hypothesis testing based method for normalization and detection of DE genes. Subsequently we carry out extensive simulation studies in Section 3. In Section 4, we evaluate the merits of our approach by applying it to a liver and kidney dataset, and demonstrate that it outperforms the alternatives. Finally, some conclusions and suggestions are made.

## Materials and Methods

### A hypothesis testing based normalization scaling factor method

We propose a new normalization procedure, called hypothesis testing based normalization (HTN), to reduce the bias of normalization by employing the available knowledge of housekeeping genes. We first introduce some notations. Let *Y*_*gk*_ be the observed count and *μ*_*gk*_ be the true expression level of gene *g* in library *k*, where *k* = 1, 2 and *g* = 1, …, *G*. The length of gene *g* is denoted by *L*_*g*_ and the total number of reads for library *k* is denoted by *N*_*k*_. Assuming the observed count is proportional to the product of the true expression level and the gene length, the expected value of *Y*_*gk*_ is formulated as

$$E\left[Y_{gk}\right]=\frac{\mu_{gk}L_{g}}{S_{k}}N_{k},\;\;\;$$ (1)

where $S_{k}=\sum_{g=1}^{G}\;\mu_{gk}L_{g}$ is the total RNA expression of sample *k*. For two samples or libraries, we test

$${\text{H}}_{0g}:\mu_{g1}=\mu_{g2}\;\;\;vs\;\;\;{\text{H}}_{1g}:\mu_{g1}\neq \mu_{g2}\;\;\;\text{for}\;\;\;\text{all}\;\;\;g.$$ (2)

Under Eq (1), the above test is equivalent to

$${\text{H}}_{0}:E\left[Y_{g1}\right]=\left(\frac{S_{2}}{S_{1}}\times \frac{N_{1}}{N_{2}}\right)E\left[Y_{g2}\right]\;\;\;vs\;\;\;{\text{H}}_{1}:E\left[Y_{g1}\right]\neq \left(\frac{S_{2}}{S_{1}}\times \frac{N_{1}}{N_{2}}\right)E\left[Y_{g2}\right]\;\;\;\text{for}\;\text{all}\;g.$$ (3)

Let *c* = *S*_2_/*S*_1_ be the scaling factor of sample 2 relative to sample 1. Assuming that the counts mapping to a gene are Poisson-distributed, that is, *Y*_*gk*_ ∼ *Pois*(*λ*_*gk*_), the test could be specified as

$${\text{H}}_{0g}:\lambda_{g1}=c\frac{N_{1}}{N_{2}}\lambda_{g2}\;\;\;vs\;\;\;{\text{H}}_{1g}:\lambda_{g1}\neq c\frac{N_{1}}{N_{2}}\lambda_{g2}\;\;\;\text{for}\;\;\;\text{all}\;\;\;g.$$ (4)

Conditioning on *Y*_*g*1_ + *Y*_*g*2_ = *n*_*g*_, we can derive that *Y*_*g*1_ follows a binomial distribution, that is,

$$P\left(Y_{g1}|Y_{g1}+Y_{g2}=n_{g}\right)=\frac{n_{g}!}{Y_{g1}!\left(n_{g}-Y_{g1}\right)!}p_{0}^{Y_{g1}}{\left(1-p_{0}\right)}^{n_{g}-Y_{g1}},$$ (5)

where *p*_0_ = *λ*_*g*1_/(*λ*_*g*1_ + *λ*_*g*2_) = (*cN*_1_/*N*_2_)/(1 + *cN*_1_/*N*_2_). The p-value for testing *H*_0*g*_ is then calculated as

$$\begin{array}{ll} p_{g}\left(c\right) & =P\left(\left|Y_{g1}-c\frac{N_{1}}{N_{2}}\left(n_{g}-Y_{g1}\right)\right|\geq \left|y_{g1}-c\frac{N_{1}}{N_{2}}y_{g2}\right|\right)\\ & =P\left(\left|\left(1+c\frac{N_{1}}{N_{2}}\right)Y_{g1}-c\frac{N_{1}}{N_{2}}n_{g}\right|\geq \left|\left(1+c\frac{N_{1}}{N_{2}}\right)y_{g1}-c\frac{N_{1}}{N_{2}}n_{g}\right|\right) \end{array}$$ (6)

where *y*_*g*1_, *y*_*g*2_ are the observed counts of gene *g* in these two samples, respectively, and *n*_*g*_ = *y*_*g*1_ + *y*_*g*2_. Note that *p*_*g*_(*c*) is a function of unknown *c*. Once *c* is determined, we could calculate the p-values for all genes and hence determine which genes are differentially expressed.

In our method, we are supposed to have a set of housekeeping genes in priori, which could be reported in published studies or selected based on certain biological information, for example, the GO terms of the genes [14]. Assume we have *m* housekeeping genes in total and denote the set of housekeeping genes as *H*. Given the true value of *c*, the p-values of housekeeping genes are supposed to follow a uniform distribution on (0, 1). Therefore given the significance level *α*, the false discovery rate of those genes is supposed to be around the nominal level if *c* is correctly specified. In other words, we find the optimal value of *c*, denoted as $\hat{c}$, by minimizing the following objective function

$$\left|\frac{1}{m}\sum_{g\in H}I\left(p_{g}\left(c\right)<\alpha |H_{0},c\right)-\alpha \right|.$$ (7)

Note that theoretically $\hat{c}$ does not depend on the chosen *α*. In practice, we observe that $\hat{c}$ is almost the same for varying *α*. To reduce the arbitrariness of choosing *α*, we set the final value of $\hat{c}$ as its mean value when *α* = 0.1, 0.2, …, 0.9.

## Simulation studies

In this section, we assess the performance of the proposed method by a number of simulation studies. To evaluate the overall effectiveness of HTN, we also compare it with recent methods, including library size, TMM [10], IMM [11], Bull [13] and NHKS [14]. Both Bull and NHKS employ the information of housekeeping genes to determine the global scaling factor. In simulation studies, we have no prior biological information of the genes. Chen et al. [14] suggested to use a statistic called coefficient of variation, which measures the stability of gene expression, to select the most stable housekeeping genes. As in their studies [13, 14], we select a single and 15 housekeeping genes for Bull and NHKS, respectively. Note that for the sample with a single point, we can not calculate its coefficient of variation statistic. Therefore, we will not compare the methods with Bull and NHKS in such case.

In the simulation studies, we generate a synthetic data according to the method described in Robinson et al. [10]. We set different values for the number of genes expressed uniquely to each sample, the proportion, the magnitude and the direction of DE genes between samples under two conditions. We randomly draw data from a given empirical distribution of real counts. We set the expectation of Poison distribution from the sampled read counts by dividing the sum *S*_*k*_ and multiplying a specified library size *N*_*k*_. With the given mean, we randomly draw data from the corresponding Poisson distribution. Some DE genes are inserted in the data, therefore, we use different statistics to rank the genes and calculate the number of false discoveries [15, 16] for each ranking. In this simulation, we consider two cases: no-repeat sample for each condition and repeat samples for each condition. In each case, we have 500 housekeeping genes by default. We replicate the simulation studies for 100 times and report the average performance of those normalization methods.

**Fig 1:**
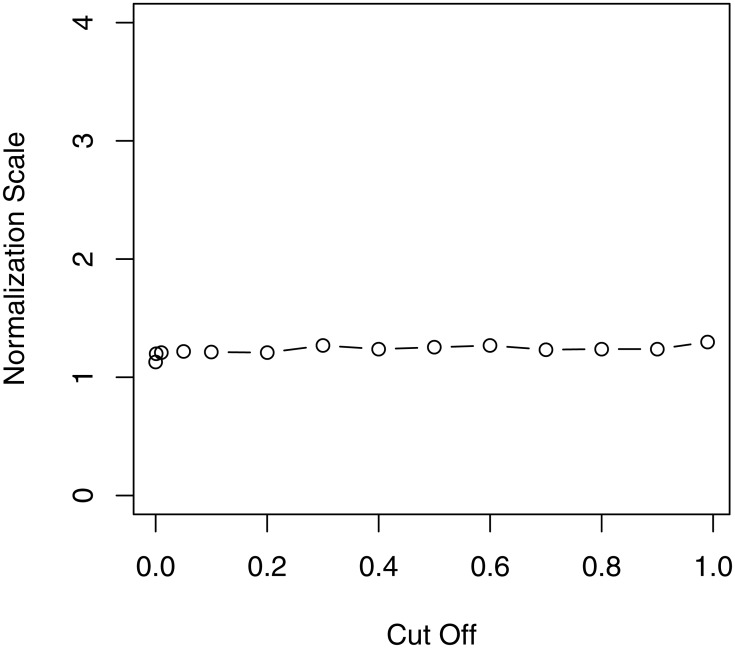
The scaling value for each p-value cutoff in Study 1.

**Fig 2:**
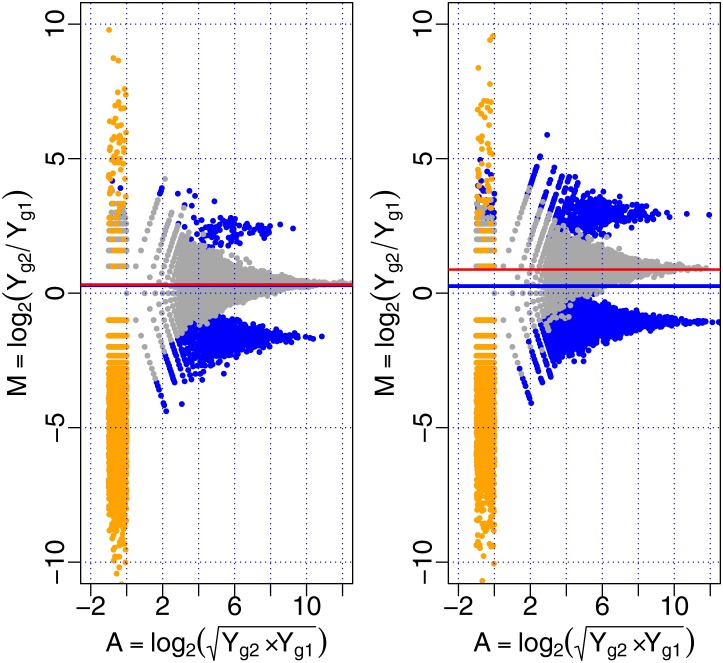
M versus A plots of different rates of DE genes. The left panel and right panel are the MA plots for DE genes at a rate of 0.1 and 0.5, respectively. The blue line is the scale of TMM normalization and the red line is the scale of HTN normalization.

In Study 1, we simulate data from only one sample for each condition. We consider two conditions and each is at a rate of 0.1 and 0.5 DE genes at a 1.5-fold level, respectively, and 90% of DE genes are higher in the second condition. In both conditions, let the expression of 10% of genes equal to zero in the first sample and the expression of the corresponding genes in the second sample not equal to zero. Fig 1 shows the scaling factor for each p-value cutoff in the simulation, which demonstrates that the scaling factor is stable for any p-value cutoff. Fig 2 shows M versus A plots for different rates of DE genes, and the scales of the HTN normalization and the TMM normalization. From the left panel of Fig 2, that two scaling factors of normalization are very close for 10% differential expression of total genes. However, as shown in the right panel of Fig 2, when the rate of differentially expressed genes increases to 50%, the red line (HTN) is much closer to the center of non-DE genes than the blue line (TMM). It suggests that HTN gives a more accurate estimate of the normalization factor in this substantive asymmetric setting, that is, for large DE rate.

**Fig 3:**
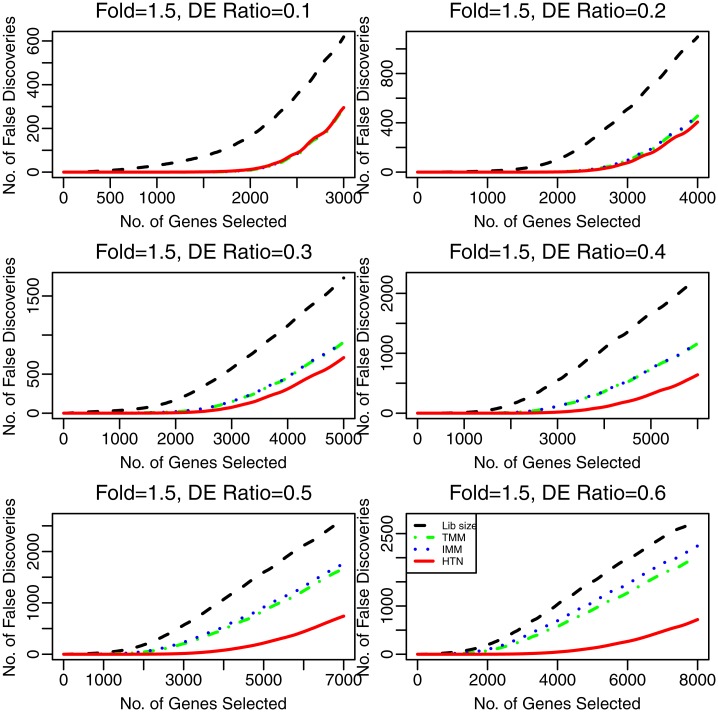
The panels are the false discovery number of test for DE genes at the rates of 0.1, 0.2, 0.3, 0.4, 0.5 and 0.6, respectively.

For no-repeat sample in Study 1, we compare the false discovery rate (FDR) of all normalization methods with different numbers of selected genes. The FDR curves are shown in Fig 3 for DE genes at the rates of 0.1, 0.2, 0.3, 0.4, 0.5 and 0.6, given 1.5-fold level, respectively. From upper panels of Fig 3, we can see that HTN, TMM and IMM have almost the same performance and they are much better than other normalization methods. However, when the rate of DE genes is larger than 0.3, HTN outperforms other methods. Therefore, we can draw the conclusion that HTN performs robustly well for varying rates of DE genes, and has better performance than other methods in the case of a large rate of DE genes.

**Fig 4:**
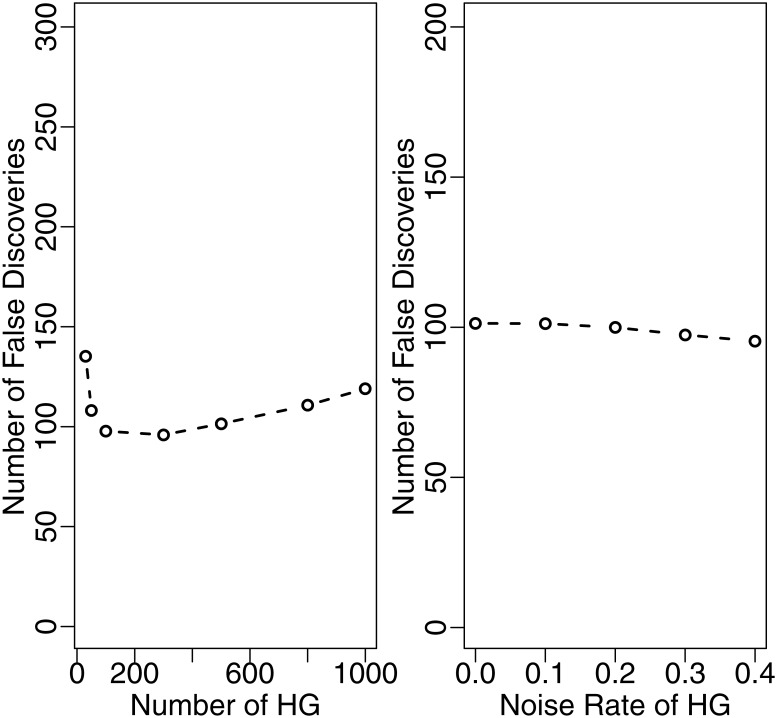
False discovery number for different numbers of housekeeping genes and different rates of noise in housekeeping genes with HTN method.

In addition, we further check the robustness of HTN with respect to the signal strength of housekeeping genes. In the above simulation setting, when the rate of DE genes equals 0.4 at a 1.5-fold level, we consider two scenarios: (1) a varying number of housekeeping genes from 50 to 1000; and (2) a varying rate of housekeeping genes that are actually DE genes, which are randomly drawn from all of DE genes. As shown in Fig 4, the numbers of false discovery genes are almost the same in those cases, indicating that HTN is indeed quite robust.

**Fig 5:**
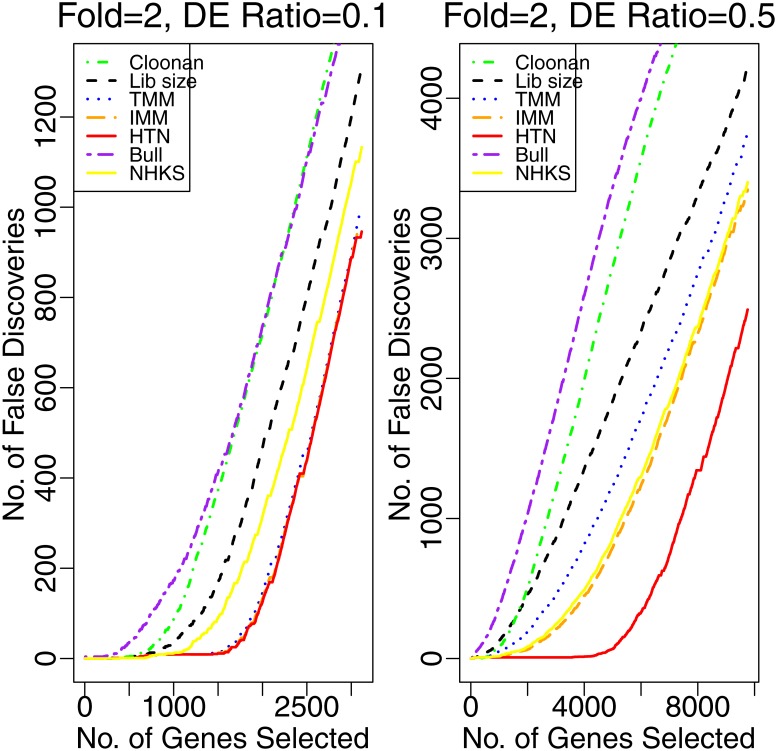
False discovery number for different normalization methods. The left panel and the right panel are the false discovery plots for DE genes at the rates of 0.1 and 0.5, respectively.

In Study 2, we consider the replicate samples for each condition with different rates of DE genes and compare the proposed method with several popular methods. Here, we consider the performance of the following methods: length-normalized count (Cloonan et al. [17]), Poisson exact test [7] with library size, TMM [10], IMM [11], Bull [13], NHKS [14] and HTN normalization. The essence of virtual length [1] and RPKM [9] are the same as library size normalization and we do not compare them here. Fig 5 shows the false discovery curves of those methods when the genes have different rates of DE genes. The left panel of Fig 5 shows that the FDRs of HTN are similar as those of TMM and IMM with Poisson likelihood ratio statistic or Poisson exact statistic, when the DE rate equals 0.1. However, as the DE rate increases to 0.5, HTN outperforms the alternatives with a lower false discovery rate.

## Application to real examples

We apply the proposed HTN method to two real data sets, including several technical replicates of a liver and kidney RNA source [5] (S1 File) and the mouse embryoid bodies versus embryonic stem cells dataset [17], and compare it with other methods. We download human housekeeping genes from [18](S2 File), which is described in [19], and then use the biomaRt package [20] in Bioconductor [21] to match them to the Ensembl gene identifiers. Robinson et al. [10] has also analyzed those real data. For the first real application, Chen et al. [14] specified 15 housekeeping genes for the liver and kidney dataset normalization in their study. Those genes are also found in the above housekeeping genes, thus we directly use them for Bull and NHKS in this example. For the second real application, given that there is no replicate data in each condition, we will not compare the methods with Bull and NHKS.

We use the exact Poisson statistic to obtain *p* − *values* by testing two different conditions and regard the genes as differentially expressed between liver and kidney if their *p* − *value* is smaller than 0.0001. Table 1 shows the number of DE genes reported by different normalization methods. From Table 1, we can see that HTN detects 8083 DE genes, 46% of which are significantly higher in liver. The total DE genes and the ratio of DE genes significantly higher in liver (or kidney) by using HTN are similar to those of TMM and IMM. Note that the library size normalization method and NHKS report a much larger number of DE genes that are significantly higher in kidney, while Bull reports more significant genes in liver, and this leads to a larger number of total DE genes in their results. For housekeeping genes, there are 330 DE genes reported by HTN, which is also similar to the results of TMM (329) and IMM (329). However, there are more DE genes out of 538 housekeeping genes for the other three normalization methods, which suggests a much larger number of false positive than that of HTN.

**Table 1:** The number of DE genes between liver and kidney at a cutoff p-value < 10^−4^ for different normalization methods.

	Library size	TMM	IMM	HTN	Bull	NHKS	Overlap
Higher in liver	2082	3759	3797	3680	7248	2836	2082
Higher in kidney	7496	4310	4273	4403	2094	5679	2083
Total	9578	8069	8070	8083	9342	8515	4165
House keeping genes (538)							
Higher in liver	39	120	123	119	287	82	14
Higher in kidney	358	209	206	211	93	276	44
Total	397	329	329	330	380	358	58

The second dataset is comparing mouse embryoid bodies versus embryonic stem cells, which is downloaded from [17], sequenced on the SOLiD system. In this dataset, there are 19005 genes in total, 495 of which are “housekeeping” genes as we know [22]. We get *p* − *value* for each gene by using the amended sage.test function [23]. Table 2 shows the results of DE genes output by different normalization methods. The number of DE genes significantly higher in EB is about 22.5%, which is much lower than that of ES (77.5%) by using the HTN normalization. There are 362 DE genes out of 495 housekeeping genes reported by HTN, which is much lower than that of library size normalization (411), TMM (397), IMM (402). Thus, based on the available knowledge of housekeeping genes, HTN tends to have a lower false discovery rate than alternatives in this case. However, as we note, HPN reports 9896 DE genes in total, which is much more than that of library size normalization (9295), TMM (9328) and IMM (9383).

**Table 2:** The number of DE genes between embryoid bodies (EB) and embryonic stem cells (ES) at a cutoff p-value < 10^−4^ for different normalization methods.

	Library size	TMM	IMM	HTN	Overlap
Higher in EB	4441	4156	3941	2227	2189
Higher in ES	4854	5172	5442	7669	4854
Total	9295	9328	9383	9896	7043
House keeping genes (495)					
Higher in EB	279	258	228	104	96
Higher in ES	132	139	174	258	132
Total	411	397	402	362	228

## Conclusion

In order to compare the genes expression and thus to detect differently expressed genes between samples, normalization is a crucial step for downstream analysis. In this paper, assuming the information of housekeeping genes is known, we propose a novel normalization method called HTN, which is based on a hypothesis testing, and show it is more effective and robust for normalizing the RNA-seq depth between different samples. The estimated scaling factors between samples can be incorporated into currently used statistical test methods for differential gene expression analysis. The knowledge of housekeeping genes is essential for using our method. To obtain housekeeping genes, users may check the relevant published studies, such as [14, 19].

In the simulation studies, we assess the performance of the proposed method by considering varying ratios of DE genes and varying signal strength of housekeeping genes. We observe that when the ratio is high, the HTN normalization method significantly outperforms the state-of-the-art methods with a lower false discovery rate. The real data analysis also shows that our new method has better performance when judging from the available knowledge of housekeeping genes. Compared with Bull [13] and NHKS [14], which also utilize housekeeping genes, our method seems to be more robust and better, at least as well as, due to that we use all housekeeping genes and the type I error statistic evaluates the overall change of the expression of housekeeping genes more efficiently. In conclusion, our empirical studies suggest that the HTN method is a competing alternative for the normalization and differential expression analysis of RNA-seq data.

## Supporting Information

S1 File This is the real data of a liver and kidney RNA source. (TXT)

S2 File This is the housekeeping genes of human. (TXT)
